# External validation of the barcelona magnetic resonance imaging predictive model for detecting significant prostate cancer including men receiving 5-alpha reductase inhibitors

**DOI:** 10.1007/s00345-024-05092-0

**Published:** 2024-07-10

**Authors:** Juan Morote, Ángel Borque-Fernando, Luis M. Esteban, Natàlia Picola, Jesús Muñoz-Rodriguez, Nahuel Paesano, Xavier Ruiz-Plazas, Marta V. Muñoz-Rivero, Ana Celma, Gemma García-de Manuel, Berta Miró, José M. Abascal, Pol Servian

**Affiliations:** 1 Department of Urology, Vall d´Hebron Hospital, Barcelona, Spain; 2 Department of Surgery, Universitat Autònoma de Barcelona, Bellaterra, Spain; 3 Department of Urology, Hospital Universitario Miguel Servet, IIS-Aragon, Zaragoza, Spain; 4 Department of Applied Mathematics, Escuela Universitaria Politécnica La Almunia, Universidad de Zaragoza, Zaragoza, Spain; 5 Department of Urology, Hospital Universitari de Bellvitge, Hospitalet de Llobregat, Spain; 6 Department of Urology, Hospital Universitari Parc Taulí, Sabadell, Spain; 7 Clínica Creu Blanca, Barcelona, Spain; 8 Department of Urology, Hospital Universitari Joan XXIII, Tarragona, Spain; 9 Department of Urology, Hospital Universitari Arnau de Vilanova, Lleida, Spain; 10 Department of Urology, Hospital Universitari Josep Trueta, Girona, Spain; 11 Unit of Statistics and Bioinformatics, Vall d´Hebron Research Institute, Barcelona, Spain; 12 Department of Urology, Parc de Salut Mar, Barcelona, Spain; 13 Department of Urology, Hospital Germans Trias i Pujol, Badalona, Spain

**Keywords:** Risk calculator, Predictive model, Significant prostate cancer, 5-alpha reductase inhibitor, Validation

## Abstract

**Purpose:**

To validate the Barcelona-magnetic resonance imaging predictive model (BCN-MRI PM) for clinically significant prostate cancer (csPCa) in Catalonia, a Spanish region with 7.9 million inhabitants. Additionally, the BCN-MRI PM is validated in men receiving 5-alpha reductase inhibitors (5-ARI).

**Materials and methods:**

A population of 2,212 men with prostate-specific antigen serum level > 3.0 ng/ml and/or a suspicious digital rectal examination who underwent multiparametric MRI and targeted and/or systematic biopsies in the year 2022, at ten participant centers of the Catalonian csPCa early detection program, were selected. 120 individuals (5.7%) were identified as receiving 5-ARI treatment for longer than a year. The risk of csPCa was retrospectively assessed with the Barcelona-risk calculator 2 (BCN-RC 2). Men undergoing 5-ARI treatment for less than a year were excluded. CsPCa was defined when the grade group was *≥* 2.

**Results:**

The area under the curve of the BCN-MRI PM in 5-ARI naïve men was 0.824 (95% CI 0.783–0.842) and 0.849 (0.806–0.916) in those receiving 5-ARI treatment, *p* 0.475. Specificities at 100, 97.5, and 95% sensitivity thresholds were to 2.7, 29.3, and 39% in 5-ARI naïve men, while 43.5, 46.4, and 47.8%, respectively in 5-ARI users. The application of BCN-MRI PM would result in a reduction of 23.8% of prostate biopsies missing 5% of csPCa in 5-ARI naïve men, while reducing 25% of prostate biopsies without missing csPCa in 5-ARI users.

**Conclusions:**

The BCN-MRI PM has achieved successful validation in Catalonia and, notably, for the first time, in men undergoing 5-ARI treatment.

**Supplementary Information:**

The online version contains supplementary material available at 10.1007/s00345-024-05092-0.

## Introduction

Prostate cancer (PCa) is the most common malignant neoplasm and the third leading cause of cancer death in men worldwide [1]. In 2009, the European Randomised Screening Prostate Cancer (ERSPC) trial reported a decrease in PCa-specific mortality in men subjected to screening compared to those of the control arm after seven years of follow-up [2]. Fifteen years later a higher than 20% decrease in specific PCa mortality remains based on the early detection and treatment of clinically significant PCa (csPCa) [3]. As consequence, the focus of early detection of PCa has evolved to csPCa. This paradigm shift has been made possible through the widespread use of prostatic magnetic resonance imaging (MRI), which is employed to select candidates for prostate biopsy and perform targeted biopsies of suspicious lesions identified as having csPCa through MRI pinpointing [4]. Despite these advancements, uncertain scenarios with excessive unnecessary prostate biopsies and overdetection of insignificant tumors (iPCa) remain. Current predictive models, based on the prostate imaging report and data system (PI-RADS) and clinical data, have emerged as leading tools to improve the efficacy of csPCa diagnostic approaches [5]. The European Association of Urology currently advocates risk-stratified pathways for csPCa screening, based on predictive models [6].

The Barcelona MRI-predictive model (BCN-MRI PM) was developed to predict individual probabilities of csPCa in 1,486 men with suspected PCa who underwent pre-biopsy multiparametric MRI (mpMRI) followed by targeted and/or systematic biopsies, and the web app BCN-risk calculator 2 (BCN-RC 2) was designed. This development was conducted at a single institution and subsequently externally validated in a cohort of 946 men from two other institutions in the metropolitan area of Barcelona. Men treated with 5-alpha reductase inhibitors (5-ARI) were excluded from the development and external validation cohorts due to their known impact on serum PSA levels and prostate volume [7]. The performance of the BCN-RC 2 was superior to that of the Rotterdam MRI-RC in the external validation cohort [8].

Symptomatic benign prostatic hyperplasia (BPH) is a highly prevalent condition at the age when PCa screening is recommended. This results in many men with suspected PCa receiving medical treatment, being 5-ARIs used for alleviating voiding symptoms and preventing disease progression [9]. It is known the effect of 5-ARIs in PCa prevention, especially in low-grade tumors [10, 11], which resulted in a relative increase in high-grade PCa detection without consequences on PCa mortality [12]. The role of 5-ARIs in preventing the progression of low-grade PCa on active surveillance is currently under discussion [13].

Our main objective is to validate the BCN-MRI PM in Catalonia, a Spanish region with 7.9 million inhabitants. Additionally, considering that some men suspected of having PCa undergo 5-ARI treatment, a secondary objective is to validate the BCN-MRI PM in this specific cohort.

## Methods

### Design, participants, and setting

This is a retrospective study for the validation of the BCN MRI-PM in 2,212 suspected PCa men who underwent mpMRI and prostate biopsy between January 1, and December 31, 2022, in ten participant centres of the Catalonian sPCa early detection programme. Among them, 120 (5.4%) were found to be receiving 5-ARI treatment for longer than one year. Twelve men undergoing 5-ARI treatment less than one year were previously excluded for the study.

### Diagnostic pathway of csPCa

SPCa early detection program of Catalonia is an opportunistic program existing from the year 2010. Serum PSA testing is offered or demanded by men to general practitioners or urologist at the primary health system, as DRE unfrequently. When serum PSA is higher than 3.0 ng/mL or an abnormal DRE is detected, men are referred to the sPCa early detection office, usually attended by the urologists who perform prostate biopsies, in hospital centers. PCa suspicion is verified by repeating serum PSA measurement, an specialized DRE performed, specific anamnesis of PCa family history and previous negative prostate biopsy and MRI requested. Finally, PCa will be detected and treatment strategy or appropriate follow up suggested. Men under special conditions, as BCRA mutations, are accepted in the sPCa early detection office, following a structured pathway.

Men suspected of having PCa are subjected to 1.5 or 3.0 Tesla mpMRI, currently reported with the PI-RADS v2.1 by experienced radiologists in each participant center. Prostate biopsy was carried out in men with suspicious lesions detected in mpMRI (PI-RADS score *≥* 3) and those with PI-RADS score *≤* 2 but having any data suggesting at high risk of PCa as PSA density > 0.15, previous negative biopsy with increased PSA, and others considered.

Two to 4-core transrectal ultrasound (TRUS)-MRI image fusion-targeted biopsies of suspicious lesions and 12-core systematic biopsies were always performed in men with PI-RADS *≥* 3. Only 12-core systematic biopsies were performed in those men with PI-RADS score *≤* 2 who were considered for a prostate biopsy. Fusion MRI-TRUS image was achieved using cognitive or commercial software techniques. Prostate biopsy procedures were performed through the transrectal or transperineal route by experienced urologists without specific criteria, besides the recommendation of transperineal route from 2019. Biopsy material was referred to each local pathology department where experienced uropathologists diagnosed csPCa when the International Society of Urologic Pathology (ISUP) grade group was two or higher [14].

### Intervention

The probability of csPCa was assessed through the BCN-RC 2, available at the web application link https://mripcaprediction.shinyapps.io/MRIPCaPrediction/. The predictive variables utilized by the BCN-RC 2 are, age (years), first degree PCa family history (no vs. yes), type of biopsy (initial vs. repeated), serum PSA level (ng/ml), DRE (normal vs. suspicious), MRI prostate volume (cc), and PI-RADS v.2.1 (1–5). The probability of sPCa was the endpoint variable of the study.

### Ethical considerations

This study received the approval from the ethical committee of the coordinating center (PRAG-02/2020), and it was support by the Instituto de Salut Carlos III and the European Union (PI2020/01666). All participants signed informed consent.

### Statistical analysis

Quantitative variables were described as median and interquartile range (25–75 percentiles), and qualitative variables as percentages. Descriptive variables were compared with the Mann-Whitney U test and the Chi-square test. Calibration of the predictive model was assessed in non-5ARI naïve men and 5-ARI users. Discrimination power was determined using the receiver operating characteristic (ROC) curves and the area under the curve (AUC) which were compared with the DeLong test. The clinical utility of the models was determined using the clinical utility curve (CUC) that explored the potential rates of missed csPCa detection and the avoidable prostate biopsies. Specificities of 100, 97.5, and 95% sensitivity thresholds were analysed. The net benefit of using the BCN-RC 2 for prostate biopsy candidate´s over biopsying men with positive mpMRI was analyzed with decision curve analysis (DCA). Significant differences were considered when the *p* value was < 0.05. Transparent reporting of a multivariable prediction model for individual prognosis or diagnosis (TRIPOD) statements were followed. Statistical analyses were computed using R programming language v.4.0.3 (The R Foundation for Statistical Computing, Vienna, Austria) and SPSS v.24 (IBM, statistical package for social sciences, San Francisco, US).

## Results

### Characteristics of the study population

The characteristics of 2,212 suspected PCa men included in this study, and those corresponding to 2,092 5-ARI naïve men and 120 who were undergoing 5-ARI treatment are summarized and compared in Table 1. The median age ranged from 68 years in 5-ARI naïve to 72 in 5-ARI users, *p* < 0.001. The median serum PSA levels were 7.3 and 8.0 ng/ml, respectively, *p* 0.107. The median prostate volumes were 53 and 69 cc, respectively, *p* < 0.001. The rates of abnormal DRE were 26.8 and 35.0%, respectively, *p* = 0.057. The rates of PCa family history were 8.1 and 4.2%, respectively, *p* 0.160. The rates of repeated biopsies were 31.4 and 36.7%, respectively, *p* 0.228.

**Table 1:** Descriptive characteristics of the overall study population and comparison according to the 5-ARI exposure

Characteristic	Overall population	5-ARI naïve	5-ARI users	*p* Value
Number of men, (%)	2,212	2,092 (94.6)	120 (5.7)	-
Median age (IQR), years	68 (62–73)	68 (62–73)	72 (68–76)	< 0.001
Median PSA (IQR), ng/ml	7.3 (5.3–11.0)	7.3 (5.3–11.0)	8.0 (5.0–13.0)	0.107
Suspicious DRE, n (%)	603 (27.3)	561 (26.8)	42 (35.0)	0.057
Repeated biopsy, n (%)	701 (31.7)	657 (31.4)	44 (36.7)	0.228
PCa family history, (%)	174 (7.9)	169 (8.1)	5 (4.2)	0.160
Median Prostate volume (IQR), cc	53 (39–75)	53 (38–74)	69 (49–97)	< 0.001
PSA density, ng/ml/cc	0.14(0.09–0.13)	0.14 (0.09–0.22)	0.12 (0.08–0.21)	0.025
3.0 Tesla mpMRI, n (%)	1,549 (70.0)	1,471 (70.3)	78 (65.0)	0.216
PI-RADS score, n (%)				
*≤* 2	335 (15.1)	320 (15.3)	15 (12.5)	0.328
3	449 (20.3)	429 (20.5)	20 (16.7)	
4	960 (43.4)	903 (43.2)	57 (47.5)	
5	468 (21.2)	440 (21.0)	28 (23.3)	
Software fusion MRI-TRUS image technique, n (%)	1,375 (62.2)	1,303 (62.3)	72 (60.0)	0.384
Transperineal prostate biopsy route, n (%)	988 (44.7)	943 (45.1)	45 (37.5)	0.104
Overall PCa, n (%)	1,402 (63.4)	1,336 (63.9)	66 (55.0)	0.052
csPCa, n (%)	986 (44.6)	935 (44.7)	51 (42.5)	0.706
iPCa, n (%)	416 (18.8)	401 (19.2)	15 (12.5)	0.072

5-ARI: 5-alpha reductase inhibitor; IQR: interquartile range; PSA: prostate-specific antigen; DRE: digital rectal examination; PCa: prostate cancer; PI-RADS: Prostate imaging-report and data system; csPCa: clinically significant PCa; iPCa: insignificant PCa

MpMRI was performed in a 3.0 Tesla scanner in 70.3% of 5-ARI naïve men and 65.0% of 5-ARI users, *p* 0.216. The distribution of PI-RADS categories in 5-ARI naïve men corresponded to PI-RADS < 3 in 15.3%, to 3 in 20.5%, to 4 in 43.2%, and to 5 in 21.0%, compared to 12.5%, 16.7%, 47.5% and 23.3%, respectively, *p* 0.328. The MRI-TRUS fusion image technique was cognitive in 45.1% of 5-ARI naïve men and 37.5% of 5-ARI users, *p* 0.384. Prostate biopsy was made through transperineal route in 62.3% of 5-ARI naïve men, and 60.0% of 5-ARI users, *p* 0.104.

Overall PCa was detected in 1,402 men (63.4%), being 986 with csPCa (44.6) and 416 (18.8%) with iPCa. Overall PCa was detected in 1,336 (63.9%) 5-ARI naïve men, and 66 (55%) 5-ARI users, *p* 0.052. CsPCa was detected in 935 (44.7%) 5-ARI naïve men and 51(42.5%) 5ARI users, *p* 0.706. IPCa were detected in 401(19.2%) 5-ARI men and 15 (12.5%) 5-ARI users, *p* 0.072.

### Calibration of the BCN-MRI predictive model in 5-ARI naïve men and 5-ARI users

Calibration curves of the BCN-MRI PM in 5-ARI naïve men and 5-ARI users are presented in Supplementary Fig. 1A and 1B respectively. A small overdetection of csPCa at low predicted probability of csPCa and a small down detection in predicted probabilities over 50% in 5-ARI naïve men is observed, being the adjustment of the calibration curve to the ideal curve slowly better in 5-ARI users.

### Discrimination ability, clinical efficacy, and net benefit of the BCN-MRI predictive model for csPCa detection in 5-ARI naïve men and 5-ARI users

**Fig. 1:**
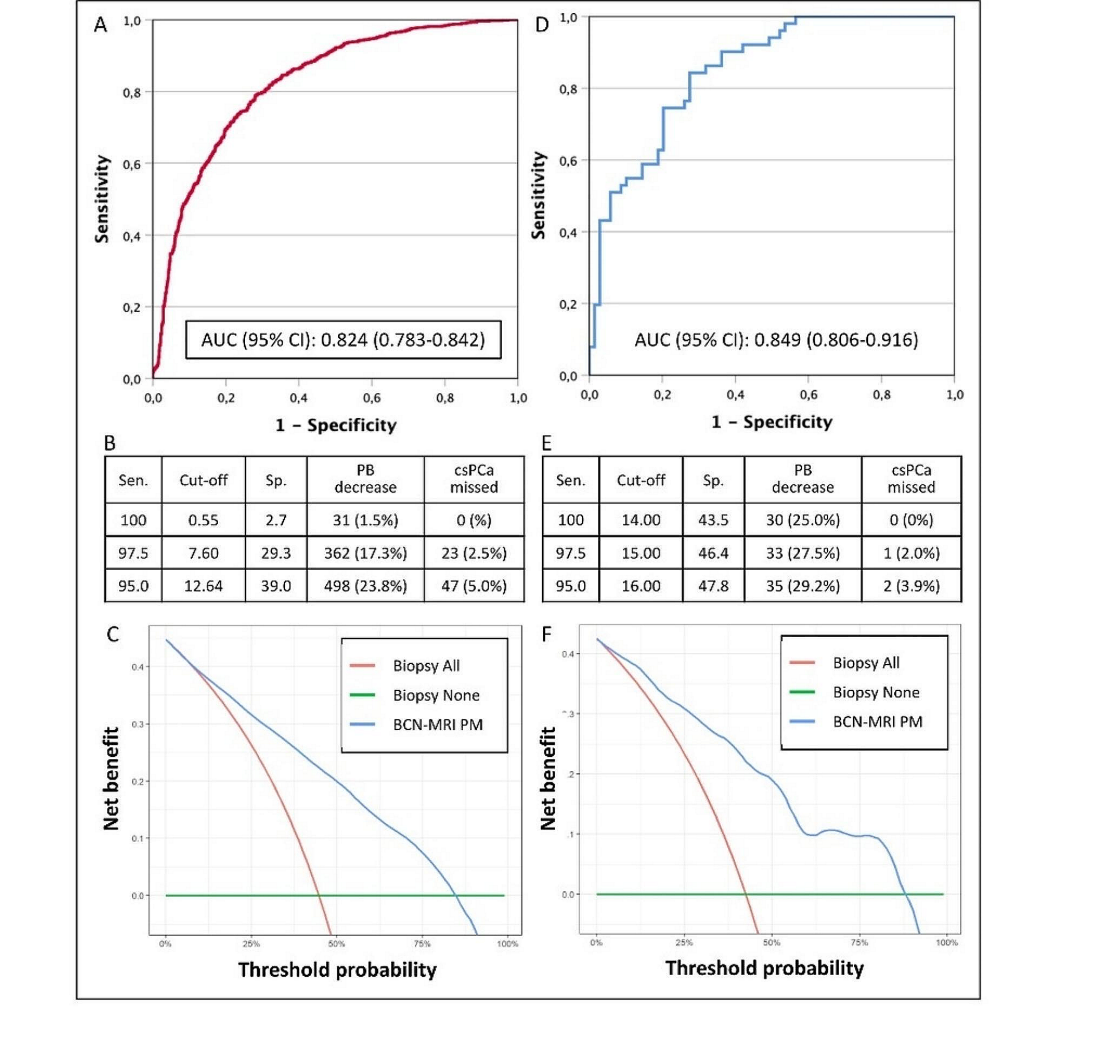
Discrimination ability, clinical efficacy, and net benefit of the BCN-MRI PM for the detection of csPCa in 5-ARI naïve suspected PCa men (**A**-**C**) and in those receiving 5-ARI treatment (**D**-**F**)

ROC curves of predicted probabilities of csPCa from the BCN-MRI PM are presented in Fig. 1A and B. The AUC in 5-ARI naïve men was 0.824 (95% CI 0.783–0.842), while 0.848 (0.806–0.916) in 5-ARI users, *p* 0.157.

Specificities corresponding to 100, 97.5, and 95% thresholds sensitivity in 5-ARI naïve men and 5-ARI users are presented in Table 1B and 1E. We note that 17.3% of prostate biopsies would be avoided using the BCN-MRI PM, missing 2.5% of sPCa in 5-ARI naïve men, while in 5-ARI users 25% of prostate biopsies would be avoided without missing sPCa. The rates of avoided prostate biopsies and its corresponding missing rate of csPCa in continuous threshold (0 to 100%) are presented in CUCs in both 5-ARI naïve men and 5-ARI users, Supplementary Fig. 2A and 3B respectively.

The net benefit of applying the BCN-MRI PM instead of biopsy all men, analyzed from DCAs performed in both 5-ARI naïve men and 5-ARI users are presented in Fig. 1C and E respectively.

The discrimination ability of the BCN-MRI PM for csPCa observed in the present validation cohort of 5-ARI naïve men and 5-ARI users is aligned with that observed previously in 5-ARI naïve men in the BCN-MRI PM development cohort and the external validation in the metropolitan area of Barcelona [7], Supplementary Table 1.

## Discussion

External validation of predictive models is mandatory in populations where they are going to be implemented [15]. The BCN-RC 2 is a user-friendly web app risk calculator designed from the BCN-MRI PM for the individual assessment of csPCa risk in men suspected of having PCa after mpMRI. This tool also has the option to select the threshold for the appropriate selection of candidates for prostate biopsy [7]. The BCN-MRI PM has been successfully validated in Catalonia, a region with 7.9 million inhabitants. Additionally, the BCN-MRI PM has been validated in men with suspected PCa receiving 5-ARI treatment for symptomatic BPH. This marks the first instance of validation for an MRI predictive model for csPCa detection in men undergoing 5-ARI treatment.

The validation of BCN-MRI PM in 5-ARI naïve men aligns with the previous validation conducted in the Barcelona metropolitan area [7]. The exclusion of twelve men with 5-ARI treatment less than a year was based on the progressive effect on serum PSA between six and twelve months, and the small number of cases [16–18]. Regarding the performance of BCN-MRI PM in men with symptomatic BPH treated with 5-ARI, we observed a calibration slightly better than that in 5-ARI naïve men. The discrimination ability of BCN-MRI PM for csPCa in 5-ARI treated men was slightly superior to that observed in 5-ARI naïve men. Using the 100% sensitivity threshold, the BCN-MRI PM was able to avoid 25% of prostate biopsies. In contrast the BCN-MRI PM could avoid 17.3% using the 97.5% sensitivity threshold and 23.8% using the 95% sensitivity threshold.

We were surprised to observe similar median serum PSA level in 5-ARI naïve men compared to those of men undergoing 5-ARI treatment. This is a shared concern aligned with the findings other studies reporting comparable serum PSA levels in 5-ARI users and 5-ARI naïve individuals at the time of prostate biopsy [16, 17]. In the recently reported PROstate Mri Outcome Database (PROMOD) study, which includes 705 men receiving 5-ARI treatment and 6,913 5-ARI naïve men, the median serum PSA levels were respectively 6.0 and 6.5 ng/ml respectively. Although a significant difference between both median values was found, the PSA serum level in 5-ARI users was notably distant from the expected half of the serum PSA level observed in 5-ARI naïve men [18]. This contrast with the recommendation for closely monitoring serum PSA level after the nadir following one year of 5-ARI treatment, and stablishing PCa suspicion based on a confirmed increase of serum PSA levels higher than 0.3 ng/ml [19]. An awareness for patients exposed to 5-ARI treatment requires a close monitoring of serum PSA level to prevent the delay in diagnosing high-grade PCa which still appears necessary [20].

The MRI in Primary Prostate Cancer after Exposure to Dutasteride (MAPPED) study was designed to investigate the radiological effects of six month’s exposure to dutasteride on low-grade PCa volume [21]. Preliminary data suggested that dutasteride is associated with an increase in the tumour apparent diffusion coefficient (ADC) and reduced visibility of the tumour on diffusion weighted imaging (DWI), without affecting T2 sequences of mpMRI [21–23]. Starobinets et al. have suggested an enhanced discrimination of mpMRI between the areas with tumours and those with benign tissue located in the peripheral zone [24]. The effect of 5-ARI exposure on the PI-RADS category and its corresponding detection of csPCa and iPCa in men with suspected PCa has been poorly analyzed [16, 17, 25]. Recently, Falagio et al. observed that exposure to 5-ARIs does not affect the association of PI-RADS score with csPCa, although a higher rate of high-grade PCa was detected in 5-ARI users with PI-RADS 5 [18].

Limitations of our study can be attributed to its retrospective design and the used criteria for defining csPCa, which may not fully reflect the final pathology through the entire prostate gland. The successful validation in the early sPCa in Catalonia, does not mean a validation in a conventional screening program. Regarding 5-ARI users it would be necessary to consider a follow up of negative biopsies. However, it´s important to acknowledge inherent constraints associated with predictive models exists. These models evaluate individual probabilities of a condition based on the characteristics of development cohort. Given that MRI is reported according to the PI-RADS score, the effectiveness of MRI predictive models should be analyzed regarding these scores to identify scenarios in which they are most useful [26]. Predictive models face challenges due to ongoing changes in the characteristics of populations where they are implemented, often necessitating recalibrations and readjustments to maintain accuracy [27]. Achieving real-time updates of predictive models poses a challenge for the future risk calculators [28]. Establishing a continuous feedback loop that involves new cases, employs appropriate machine learning algorithms, and utilizes federated networks can pave the way for risk calculators that undergo continuous updates at each participant site, ensuring their ongoing accuracy [29].

## Conclusions

The BCN-MRI PM, developed for the individual assessment of csPCa probability in prostate biopsy of suspected PCa men has undergone successful validation in the population of Catalonia. Moreover, this model has also been validated in men suspected of having PCa who undergo 5-ARI treatment due to symptomatic BPH. The BCN-MRI PM is deemed ready for use in all men with suspected PCa in Catalonia.

## Electronic supplementary material

Below is the link to the electronic supplementary material.

Supplementary Material 1

## Data Availability

The data presented in this study are available on request from the corresponding author.
