# Supplementary material for: External validation of the barcelona magnetic resonance imaging predictive model for detecting significant prostate cancer including men receiving 5-alpha reductase inhibitors
Source: World J Urol. 2024 Jul 10;42(1):393. doi: 10.1007/s00345-024-05092-0 (PMC11236874; doi:10.1007/s00345-024-05092-0)
Supplement: Supplementary file 1 — Supplementary Material 1 [file 345_2024_5092_MOESM1_ESM.docx]

Supplementary Fig.1 Calibration curves of the BCN-MRI PM in 5-ARI naïve suspected PCa men (A), and those receiving 5-ARI treatment (B)

Supplementary Fig. 2 Clinical utility curves showing the rates of saved biopsies and corresponding rates of undetected csPCa according to the threshold probability points of the BCN-MRI PM in 5-ARI naïve suspected PCa men (A) and those undergoing 5-ARI treatment (B)

Supplementary Table 1 Comparison of the discrimination ability and clinical parameters of efficacy for csPCa detection of the BCN-MRI PM in its development cohort, and the validation cohort in the metropolitan area of Barcelona, with those observed in the Catalonian validation in 5-ARI naïve suspected PCa men, and in those undergoing 5ARI treatment

| Type of Cohort | n | % sPCa detection | AUC (95% CI) | Threshold (%) | Specificity at 95% Sensitivity | % PB decrease | % sPCa missed |
| --- | --- | --- | --- | --- | --- | --- | --- |
| Development | 1.486 | 36.9 | 0.897 (0.880-0.914) | 15 | 55.7 | 40.1 | 5.4 |
| MPA Validation | 946 | 40.8 | 0.858 (0.833-0.883) | 14 | 32.3 | 39.9 | 5.0 |
| CAT Validation | 2.092 | 42.5 | 0.824 (0.783-0.916) | 13 | 39.0 | 23.8 | 5.0 |
| 5ARI Validation | 120 | 44.7 | 0.849 (0.806-0.842) | 16 | 47.8 | 29.2 | 3.9 |

n =number; sPCa =significant prostate cancer; AUC =area under the curve; CI =confidence interval; PB =prostate biopsies; Barcelona metropolitan area; CAT = Catalonia; 5ARI =5-alpha reductase inhibitors.
